# Unpredictable refeeding syndrome with severe hypophosphatemia in borderline personality disorder comorbidity: A case report

**DOI:** 10.1097/MD.0000000000034103

**Published:** 2023-06-23

**Authors:** Kazumasa Hamada, Kenichiro Sagiyama, Ryusei Nishi, Takamasa Fukumoto, Ryuichi Kato, Yuuki Fuku, Haruka Amitani, Akihiro Asakawa

**Affiliations:** a Department of Psychosomatic Internal Medicine, Kagoshima University Graduate School of Medical and Dental Sciences, Sakuragaoka, Kagoshima, Japan.

**Keywords:** boderline personality disorder, case report, hypophosphatemia, refeeding syndrome

## Abstract

Refeeding syndrome (RS) is a fatal condition caused by rapid calorie intake during starvation. Self-neglected fasting in psychiatric disorders is associated with RS. However, overeating resulting from circumventing the clinician’s instructions does not have a reportedly high risk of RS.

**Patient concerns:**

A 47-year-old undernourished woman with borderline personality disorder was hospitalized for nausea, vomiting, and diarrhea.

**Clinical findings:**

She had not eaten much for 10 days and had lost weight (56.5–51.1 kg) over 3 weeks. No abnormalities were indicated on physical examination and imaging examinations.

**Diagnoses, interventions, and outcomes:**

Infectious diseases and malignancies were excluded from the differential diagnosis. On the third day of admission, the patient’s serum phosphorus level significantly decreased to 0.7 mg/dL, and additional sodium phosphate was administered intravenously. On the fourth day, despite our instructions, the patient was found to be eating nonhospital food from the first day of admission. In conjunction with her history, a final diagnosis of RS was made. After appropriate treatments, the patient was discharged on the 15th day of hospitalization. The patient’s nausea, vomiting, and diarrhea were improved.

**Lessons:**

When undernourished patients have psychiatric disorders, including borderline personality disorder or schizophrenia, the occurrence of RS should be considered based on the patients’ poor adherence to physicians’ instructions.

## 1. Introduction

Borderline personality disorder (BPD) is a severe mental disorder with 9 characteristics: fear abandonment, unstable relationships, unstable self-image, impulsivity, self-harm, mood instability, feelings of emptiness, inappropriate anger, and dissociation/transient paranoid ideation, according to the Diagnostic and Statistical Manual of Mental Disorders, fifth edition.^[1,2]^ Diagnosing BPD requires the fulfillment of 5 of these 9 criteria, and its severity increases with the number of fulfilled criteria.^[2]^ Some reports have suggested that personality disorders, including BPD, contribute to a higher mortality rate than that of healthy individuals.^[3]^

Refeeding syndrome (RS) also has a group of life-threatening changes that can lead to death owing to the rapid caloric intake during starvation. The historical definition of RS involves various changes in metabolism and electrolytes resulting from excessive caloric intake after a course of insufficient caloric intake.^[4]^ The changes in electrolytes include hypophosphatemia, hypokalemia, and hypomagnesemia. The American Society for Parenteral and Enteral Nutrition has stated that electrolytes are equally important in RS. The reason is that the diagnosis of RS is biased toward phosphorus as physicians associate low serum phosphate with RS easily,^[4]^ though the cause of hypophosphatemia is less than that of hypokalemia. However, Skipper conducted a systematic review of 27 cases and suggested that only hypophosphatemia is consistent with RS among the reviewed data of 63 patients.^[5]^ Therefore, although hypophosphatemia is not a high occurrence,^[6]^ phosphorus may be the primary electrolyte to consider RS. To date, the main focus of RS has been on eating disorders, feeding difficulties in older adults, and severely malnourished adults with poor conditions such as cancer.^[4]^ Mental health disorders exacerbate self-neglect and result in malnourishment from their willingness to eat nothing, elevating RS risk.^[4]^ Among these patients, careful nutrition therapy can decrease the risk of RS. However, malnourished, self-neglecting patients do not always eat anything. Herein, we report the case of a patient with severe hypophosphatemia due to unpredictable RS and BPD comorbidity, despite appropriate calorie restriction during hospitalization.

## 2. Patient information and clinical findings

Written informed consent was obtained from the patient. A 47-year-old woman was admitted to our ward with postprandial nausea, vomiting, diarrhea, and drowsiness.

She had completed a master’s program at a graduate school. She frequently changed her workplace after graduation, suggesting unstable relationships, impulsivity, mood instability, and feelings of emptiness. After her divorce from her husband, she developed depression and insomnia, and she caused a self-inflicted injury to her wrist. She was hospitalized in our ward for 2 months for this. Two years later, she developed depression and insomnia again, which was triggered by a breakup with her boyfriend. She was again admitted to our ward. She was hospitalized twice within the 2 following years for anxiety about her familial relations. A year after the last admission she was involved in a traffic accident that resulted in property damage. After police questioning, she called her physician to obtain a document to prove that she did not have a psychiatric disorder. At this time, she developed diarrhea, depression, and insomnia, for which treatment was initiated 2 months later. A month later, the diarrhea worsened during a problematic relationship with another partner, and she also developed postprandial nausea and vomiting.

A few years ago, she had taken the Minnesota Multiphasic Personality Inventory. The L and K scale scores were distributed between 35 and 50 and the *F*-scale score was ≥70, indicating that she may intentionally worsen her condition by falsely claiming or exaggerating her symptoms. In addition, scores on scales 8, 4, and 6 were particularly high, which suggested the possibility of personality disorders with psychotic behaviors. She was finally diagnosed with BPD.

Upon admission, she admitted that she had not eaten much for 10 days and had lost weight (56.5–51.1 kg) over 3 weeks. Her height was 157.0 cm and her weight was 51.1 kg (body mass index [BMI], 20.7). Her body temperature was 36.5°C, and no abnormalities were indicated on physical examination. The laboratory examination results upon admission to our ward are shown in Table 1. Chest and abdominal X-rays showed no significant abnormalities. Infectious diseases and malignancies were excluded from the differential diagnosis.

**Table 1:** Laboratory results during hospitalization.

Laboratory test	Normal range	Day 1	Day 2	Day 3	Day 4	Day 5	Day 7	Day 9	Day 13
Phosphorus	2.7–4.6 mg/dL	1.3	1.9	0.7	1.2	1.7	2.3	2.6	2.6
Ca	8.8–10.1 mg/dL	7.8	7.9	8.2	8.0	8.0	8.4	8.2	8.5
BUN	8.0–20.0 mg/dL	8.2	15.9	17.2	11.9	12.3	11.4	14.6	13.8
Creatinine	0.46–0.79 mg/dL	0.87	0.70	0.60	0.60	0.58	0.65	0.69	0.76
TP	6.6–8.1 g/dL	5.8	–	6.1	–	–	–	5.9	6.2
Albumin	4.1–5.1 g/dL	4.1	–	4.2	–	–	–	–	4.2
Mg	1.8–2.3 mg/dL	2.7	–	1.7	2.1	2.0	–	2.4	–
Na	138–145 mmol/L	140	140	141	142	141	140	140	140
K	3.6–4.8 mmol/L	4.2	4.4	4.2	4.3	4.5	4.5	4.8	5.1
Cl	101–108 mmol/L	105	106	107	109	110	106	106	106
AST (GOT)	13–30 U/L	10	9	10	9	11	10	9	9
ALT (GPT)	7–23 U/L	9	8	10	9	10	10	8	7
γ-GTP	9–32 U/L	9	–	9	9	9	–	10	9

ALT (GPT) = alanine transaminase (glutamic pyruvic transaminase), AST (GOT) = aspartate transaminase (glutamic oxaloacetic transaminase), BUN = blood urea nitrogen, Ca = calcium, Cl = chlorine, γ-GTP = γ-glutamyl transpeptidase, K = potassium, Mg = magnesium, Na = sodium, TP = total protein.

## 3. Diagnostic assessment and therapeutic intervention

**Figure 1.**
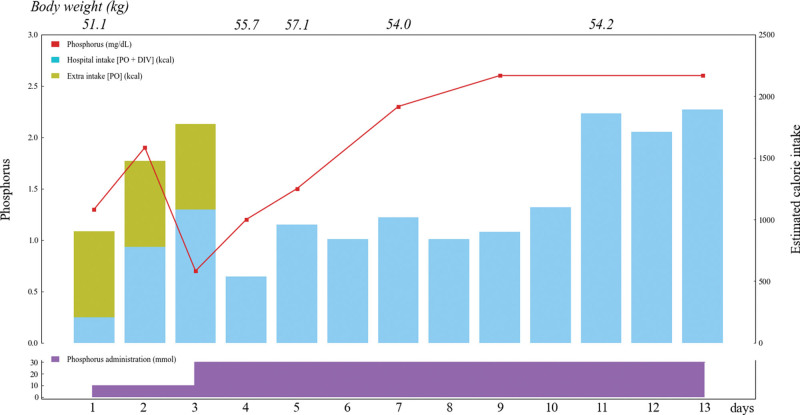
Serum phosphorus levels and estimated calorie intake during hospitalization. The red line indicates the patient’s serum phosphorus level (mg/dL). The light blue bar indicates the patient’s estimated oral and intravenous calorie intake. The light-green bar indicates the patient’s calorie intake other than hospital intake. The patient’s body weight is indicated on the graph. Finally, intravenous phosphorus administration is indicated by the purple area. DIV = intravenous drip, PO = per OS.

From the first day of hospitalization, parenteral nutrition (BFLUID® [Otsuka Pharmaceutical Factory Inc., Japan], 1000 mL/day [420 kcal/day]) was administered intravenously. At this time, we did not consider the patient to be at a high risk of RS, and we administered conventional nutrition therapy but did not allow excessive calorie intake to prevent RS. Therefore, the patient was instructed not to eat anything other than the hospital food, which was started at 900 kcal/day. The blood phosphorus concentration was 1.3 mg/dL at admission and improved to 1.9 mg/dL on the second day. However, it significantly decreased to 0.7 mg/dL on the third day (Table 1, Figure 1).

On the fourth day of admission, despite our instructions, the patient was found to be eating nonhospital food (pasta equivalent to 500 kcal and beverage equivalent to 195 kcal) from the first day of admission. The blood phosphorus concentration significantly decreased. In conjunction with her history, a final diagnosis of the RS was made.

Besides 10 mmol/day of phosphate from BFLUID® (1.002 g of dibasic potassium phosphate and 1.542 g of dibasic sodium phosphate hydrate), intravenous sodium phosphate solution at 20 mmol/day was added from the third day. Subsequently, the blood phosphorus concentration improved to 2.3 mg/dL on the seventh day (Fig. 1).

## 4. Outcomes

Nausea, vomiting, diarrhea, and drowsiness improved, and the patient was discharged on the 15th day of hospitalization.

## 5. Discussion

The incidence of RS has been reported in some case series^[4]^; however, it remains unclear owing to the lack of a precise definition of the syndrome caused by the insufficient robustness of epidemiological research.^[7]^ A recent literature review suggests that the National Institute for Health and Clinical Excellence criteria are needed to prevent and stratify the risk of RS.^[8]^ However, RS screening has various other criteria besides National Institute for Health and Clinical Excellence. There is no universal method to safely advance nutritional administration.^[4]^

As described in the present report, we did not consider the patient to be at high risk for RS. Currently, the risk of RS cannot be quantified, and the American Society for Parenteral and Enteral Nutrition consensus suggests that the definition of “mild risk” is inappropriate in adults because mild RS risk may lack clinical significance, and defining mild RS may lead to overdiagnosis.^[4]^ Therefore, moderate and significant risk criteria are defined. Although the definition is incomplete and the importance of each factor is unknown, in our case, the patient’s BMI was 20.7, weight loss was 9.5% within 3 weeks, negligible oral intake for 10 days. In addition, the patient’s risk-elevating factor was only prolonged fasting, which was not as severe as cancer or acquired immunodeficiency syndrome. Therefore, we conclude that this patient was not at significant risk of RS but needed careful consideration for daily calorie intake from both enteral and parenteral nutrition therapy from the first day of admission (Fig. 1). However, as mentioned in the present case report, the patient was found to take additional caloric intake, which increased the estimated caloric intake by twice than planned nutrition therapy. The severe serum phosphorus level drop on day 3 is consistent with the acute calorie increase on the previous day.

Mental health disorders may elevate the risk of RS from poor self-nourishment. Hershkowitz et al have reported a 25-year-old woman’s RS in schizophrenia.^[9]^ Her BMI on admission was 12.5, and her body weight had decreased significantly over the past year. Phosphorus concentrations were not measured upon admission. The “start low, go slow” strategy^[10]^ was utilized to avoid the anticipated risk of RS. Therefore, the approved caloric intake had been restricted, and her family or nurse carefully observed her eating to achieve the feeding plan. On the second day, because she was free to eat by herself because of her strong willingness, she circumvented the instruction and consumed twice as many calories as permitted. The next day, she developed severe hypophosphatemia and RS. This reported case is similar to our case in patients’ deviation from clinicians’ instruction. Although self-neglecting-based fasting is the main risk factor for RS in patients with mental health disorders, overeating resulting from deviation from nutritional therapy may also be considered a risk factor for RS. To our knowledge, our case is the first BPD patient to develop RS from overeating, resulting from circumventing the clinicians’ nutrition therapy.

In conclusion, when undernourished patients have psychiatric disorders including BPD or schizophrenia, the occurrence of RS should be considered from patients’ poor adherence to physician’s instructions.

## Author contributions

**Conceptualization:** Kenichiro Sagiyama, Ryusei Nishi, Haruka Amitani, Akihiro Asakawa.

**Data curation:** Kazumasa Hamada.

**Investigation:** Kazumasa Hamada, Ryusei Nishi, Takamasa Fukumoto, Ryuichi Kato, Yuuki Fuku.

**Supervision:** Kenichiro Sagiyama, Haruka Amitani, Akihiro Asakawa.

**Writing – original draft:** Kazumasa Hamada.

**Writing – review & editing:** Ryusei Nishi, Haruka Amitani, Akihiro Asakawa.
